# The Precursors Used for Developing Geopolymer Composites for Circular Economy—A Review

**DOI:** 10.3390/ma17071696

**Published:** 2024-04-07

**Authors:** Gabriel Furtos, Doina Prodan, Codruta Sarosi, Dorin Popa, Marioara Moldovan, Kinga Korniejenko

**Affiliations:** 1 Raluca Ripan Institute of Research in Chemistry, Babes Bolyai University, 30 Fantanele Street, 400294 Cluj Napoca, Romania; codruta.sarosi@ubbcluj.ro (C.S.); marioara.moldovan@ubbcluj.ro (M.M.); 2 Faculty of Economic Sciences, 1 Decembrie 1918 University of Alba Iulia, 15–17 Unirii Street, 510009 Alba Iulia, Romania; dorinn2005@yahoo.com; 3 Faculty of Materials Engineering and Physics, Cracow University of Technology, 31-864 Cracow, Poland; kinga.korniejenko@pk.edu.pl

**Keywords:** fly ash, geopolymer composites, cement, fibers, circular economy, SEM morphology

## Abstract

Considering recent climate changes, special importance is given to any attempt to depollute and protect the environment. A circular economy seems to be the ideal solution for the valorization of mineral waste, resulting from various industrial branches, by reintroducing them in the process of obtaining alternative building materials, more friendly to the environment. Geopolymers can be considered as a promising option compared to Portland cement. Information about the influence of the composition of the precursors, the influence of the activation system on the mechanical properties or the setting time could lead to the anticipation of new formulations of geopolymers or to the improvement of some of their properties. Reinforcement components, different polymers and expansion agents can positively or negatively influence the properties of geopolymers in the short or long term.

## 1. Introduction

**Figure 1:**
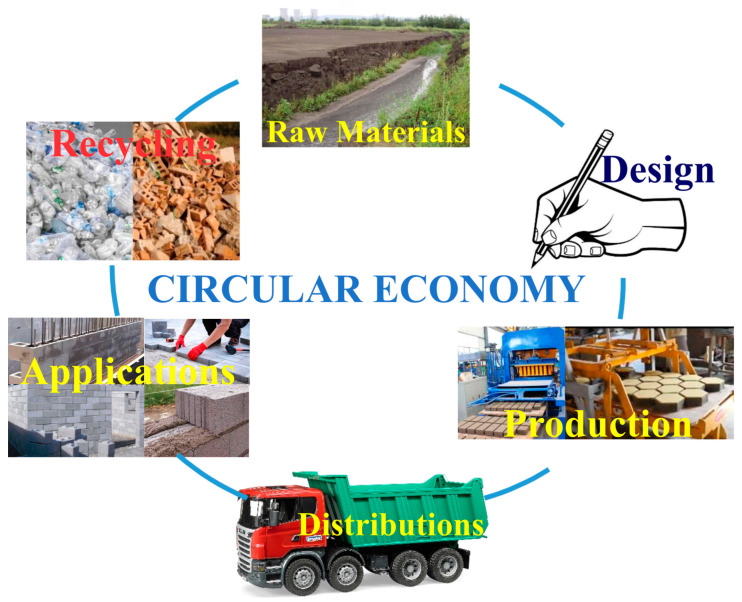
The stages of the circular economy.

The exhaustion of natural resources, the degradation of the environment, the large emissions of CO_2_ into the atmosphere, and the rising deterioration of the environment have fueled the need for urgent solutions to reduce the negative effects of pollution in order to slow global warming [1]. In this view, the concept of circular economy appeared [2,3], which can provide an option for reusing large amounts of waste, encouraging their reuse in the manufacturing circuit, through innovative methods, to obtain new building materials. Coal represents an important resource for obtaining electricity. However, the coal-burning process produces significant amounts of waste, such as slag and fly ash [2,3]. Improper storage of these wastes can lead to air, soil, or water contamination, leading to a negative impact on the environment. In addition to this waste, there are other examples like glass [4] and bauxite waste [5]. Figure 1 shows the main stages of the circular economy.

The geopolymers introduced by Davidovits [6] are materials based on aluminosilicate precursors with low calcium content and alkaline activation. Davidovits [6] relies on the fact that after the geopolymerization process, a zeolitic material will be obtained, with properties such as hardness, longevity, and thermal stability similar to those of natural rocks. These materials showed good resistance to chemical corrosion, superior mechanical properties, and good durability [7]. On the other hand, the above-described materials are more correctly called alkali-activated materials because they are a combination of cement hydrates and geopolymeric components. The conversion of aluminosilicate precursors can be improved by increasing their reactivity to geopolymerization depending on the type and ratio of the alkaline activators used [8,9]. 

Zhuang et al. [10] admitted that an important factor that can influence the geopolymer’s properties is the active substance Si/Al molar ratio. The alkaline solution could be another important factor that could influence the properties of the geopolymer. The surface charge density (inversely proportional to the radius of the cation) influences the degree of polymerization of the geopolymer; the lower the charge density, the higher the degree of polymerization. A higher temperature [10] also strengthens the geopolymer because the dissolution of the ash takes place at a lower temperature, and increases the setting time of the geopolymer. 

Recent research in the field of geopolymers aims to improve mechanical properties, especially crack resistance, by introducing reinforcement components such as fibers. Vegetable fibers are cheap, are found in abundance, show low density, have high specific resistance and no toxicity, and are can be used successfully in building materials [3].

All countries face major problems in the management of large amounts of mineral waste (fly ash, metakaolin, etc.). This could be solved by reintroducing them into a circuit to obtain alternative building materials. Data on the properties of geopolymers in the literature vary based on the origin of the precursors and the methods/parameters used to obtain them. The literature data generally only refer to geopolymers based on one or two wastes that sometimes lead to the improvement of some properties of the material. However, it is necessary to find new solutions to increase the longevity of the material, by adding some reinforcement components or some additives.

This review provides information about the influences of some compounds (precursors, alkaline activator components, reinforcement components) and their concentrations on the material properties, in order to explain the geopolymerization process.

## 2. Geopolymers and Their Most Common Precursors

Geopolymers are based on aluminosilicate sources with high reactivity and alkaline activators. The alkaline activator is usually a combination of silicate and sodium hydroxide mixed in a wet state [1]. Liquid hardeners are usually added to obtain a geopolymeric gel that helps strengthen the geopolymer. Fly ash [11,12,13], furnace slag [14] (richer in Si), metakaolin [15] (richer in Al) [16], and glass waste are examples of the precursors that form the basis of geopolymers. Geopolymers are like zeolites, but, unlike zeolites, which show a crystalline structure, they have an amorphous structure. If the crystalline network of zeolites is formed at more than 100 °C and at a pressure of approximately 200 kPa, the crystallinity stage is not reached for the geopolymers at these parameters [17]. The choice of precursors is made according to their availability, type of application, and cost. Amorphous precursors are desired, with a reactive glassy content and a a low water requirement, which can easily release aluminum [18]. It was found that the smaller the particles of the precursors, the faster the reaction takes place and the time required for heating is considerably reduced. Also, the material’s workability and compression resistance are improved. Water sorption can be considerably controlled by particle size distribution, and the formation of the aluminosilicate gel is influenced by the amorphous filling of Al_2_O_3_ and SiO_2_, which can lead to the development of a denser network, resistant to chloride attack. A study on the influence of the particle size of class F fly ash on the compressive strength reports that the highest values were obtained for the samples with the smallest particles and with the highest activator/fly ash ratio [19].

### 2.1. Fly Ash

According to ASTM C 618 [20], fly ash is classified into type C (rich in Ca) and type F (low in Ca). Class F ash results from the burning of bituminous coal and anthracite, with less than 10 wt.% Ca, quartz, aluminosilicate glass magnetite, and mullite, which provide a pozzolanic nature and very weak cementing properties, indicated for concrete. Class C of fly ash results from the burning of sub-bituminous coals and lignite, with a higher content of Ca (up to 15 wt.%), reactive alumina and silica, showing a capacity for self-cementation in addition to the pozzolanic properties. Type C ash has hydraulic properties and swells in the presence of water. F-type ash is recommended in the synthesis of geopolymers. Therefore, the fly ash is chosen according to the suitability of the obtained material [21].

**Figure 2:**
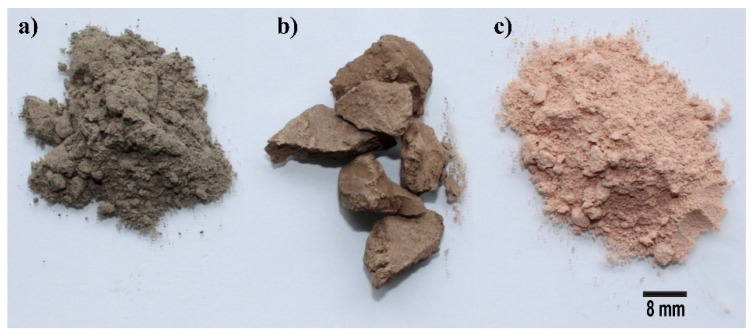
The photographs of: (**a**) class-F fly ash powder, (**b**) kaolinite and (**c**) metakaolin.

**Figure 3:**
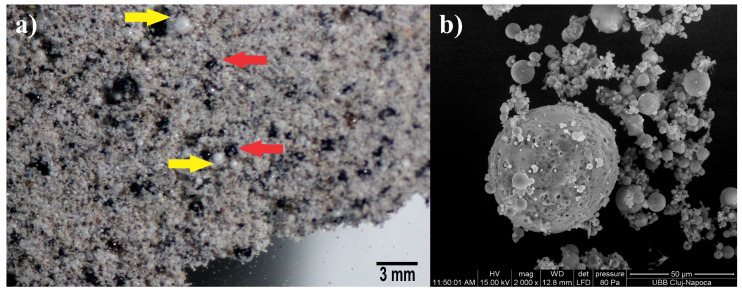
Fly ash powder (**a**) optical microscopy; (**b**) SEM of fly ash powder [12,13].

Coal from power plants burns very quickly, and the residue is rich in silica, aluminum, and iron. After melting and rapid cooling, the material solidifies in a spherical form, which is called fly ash (Figure 1). Some researchers [12,13] showed that the fly ash powder (Figure 2a) has some spherical shaped particles (Figure 3a,b). Some of these particles can be black (Figure 3a yellow arrow or white in Figure 3a red arrow) and were found to be mullite (3Al_2_O_3_·SiO_2_) and hematite (Fe_2_O_3_) [12,13], and the vitreous phase was found together with the crystalline phase [12,13]. Fly ash is used as a precursor or as a binder, especially to obtain geopolymeric cement [12,13].

The chemical composition of fly ash differs depending on the method of production or combustion temperatures (800–1800 °C) [11]. Studies show that to ensure the appropriate alkalinity, the Si/Al ratio must be between 2 and 3.5, the percentage of Fe_2_O_3_ must be below 10 wt.%, and active Si should be above 40 wt.%. The particle size below 45 µm could also lead to obtaining a geopolymer with improved properties. The concentration of Ca must not be higher than 5 wt.% because it can affect the reactivity of Si and Al [6,11]. For geopolymeric binders, studies regarding fly ash [22], an activation process with alkalis that leads to depolymerization of the precursors, followed by the repolymerization of disintegrated monomers that form aluminosilicate networks with the role of improving the properties of geopolymeric composites, is needed. These activators can be obtained from both chemical reagents and high-pH waste [23]. For other types of fly ash, sulfate may be a better activator [24]. Fernández-Jiménez and Palomo [25] think that silicon has the most important role in the production of the initial gel based on zeolitic nuclei, leading to a dissolution that releases the first monomers, resulting in a gel rich in silica. Gel formation is faster when the silicate contains more dimers. 

The more aluminum in the aluminosilicate source (at least 20 wt.%), the higher the reactivity [21]. If there is less aluminum, once it is released, it is quickly consumed, and the source is no longer reactive. The silicon-rich gel becomes more stable with the penetration of dissolved Al into its structure. Sodium can stabilize the gel by balancing the aluminum monomers or be used for mixtures with low Si/Al ratios. Sodium plays the role of a load balancer, able to enter the pores of the mixtures and bind to oxygen and water molecules [21,25]. 

### 2.2. Metakaolin

Kaolin is a rock that contains minerals such as kaolinite, quartz impurities, and mica. Kaolinite is formed by a layer of silicon tetrahedra and another layer of alumina octahedra linked by hydroxyl groups. After the heating treatment, kaolinite (Figure 2b) transforms into metakaolin (Figure 2c), which has an amorphous structure, representing a source of very reactive aluminosilicates [21]. Metakaolin is one of the first precursors used in geopolymer research. It was originally used as a filler in the paper and plastic industry. Recently, various types of metakaolin have been obtained for different applications. In addition to SiO_2_ and Al_2_O_3_, metakaolin also contains a small percentage of metal oxides. In their study, Davidovits et al. [26] investigated several types of metakaolin from an exothermic point of view, recording for each type, the minimum time in which the thermal maximum is reached in an alkaline solution. Solouki [11] reports a mixture of activated alkaline foam, with 70 wt.% tungsten waste and only 10 wt.% metakaolin as precursors. With the addition of Al powder, a higher resistance was reported compared to samples based on fly ash and metakaolin. In another study [27], soluble silicon reacted with part of the residues from a vanadium mine, while the unreacted part played the role of an aggregate.

### 2.3. Blast Furnace Slag 

The blast furnace slag obtained from the iron manufacturing process has a rich content of aluminum, magnesium, and calcium silicates. Air-cooled slag is not suitable to produce geopolymers. The blast furnace slag appears to be a precursor for obtaining geopolymers in many studies [28,29]. The structure of the slag based on aluminosilicates can be affected by the degree of polymerization or depolymerization of the network based on silica tetrahedron units, but also by the addition of certain cations or the physical state of the slag (crystalline, glassy, or liquid) [30]. Blast furnace slag has a rich calcium content. This can be considered the precursor of alkaline activated cements due to the hydraulic modulus expressed by the CaO/(SiO_2_ + Al_2_O_3_) ratio greater than 1. This modulus has a direct influence on the alkaline activation of the slag. However, it was found that if the CaO/SiO_2_ ratio is over 1.50, the alkaline activation is reduced. Also, the addition of magnesium can increase the alkaline activation reaction. Unlike the geopolymer gel from geopolymers, in the case of blast furnace slag, calcium and silicate hydrates can form as secondary products. Due to the compounds with Ca, an increased strength of the cement can be obtained at an early age. However, this resistance differs depending on the composition of the slag, the method of hardening or the activator used. It has been reported that an increase in the concentration of Na_2_O in the activator solution leads to the development of tobermorite and hydrotalcite crystals and the compressive strength increases to 39.92 MPa, for a concentration of 15% Na_2_O [31]. In their study, Perná and Hanzlíček [32] report that the use of blast furnace slag as a precursor, in addition to clay, can lead to an increase in the setting time of geopolymers. The mixing method also has an important impact on the setting time. Another conclusion of the study was that, by increasing the slag content, the setting time can be substantially reduced. One study [33] reported that cement with basic slag led to a higher compressive strength than cement with acid slag. According to the standard ENV 197–1:1992 [34], the ratio between the mass of CaO and MgO and the mass of SiO_2_ must be greater than 1.0 because otherwise, the slag would be hydraulically inactive [35]. In another study, Escalante-Garcia et al. [36] found that slag with a higher glass fraction was more reactive than slag with a lower glass fraction. Liu et al. [33] reported that an increase in the glass mass content does not lead to an increase in the compressive strength. Depending on the size of the particles, some researchers [37] said that particles <5 μm ensure adequate hydration and a particle size > 20 μm provides a less reactive slag. 

### 2.4. Glass Waste

Glass waste is a rich source of silica (71–75%) and adding it to concrete improves its microstructure. The optimal size of the glass particles, approximately 38–75 µm, increases the pozzolanic activity and the development of hydration products. The durability of concrete is influenced by the pore network. The concrete based on waste glass is denser, due to the filling effect given by the glass particles that disturb the connectivity of the pores and the penetration of water and chlorides is less than for Portland cement. The sulfates attack, especially the Mg sulfate, can degrade the hydration products, causing their decalcification and leaching. The durability of concrete increases by adding glass powder with a high pozzolanic content because the calcium hydrate transforms into calcium-silicate hydrate. Geopolymerization is influenced by the Si/Al ratio, which is considered optimal between 3.3–4.5. To ensure a high solubility of glass waste in concrete, an alkaline solution at a pH of over 10.7 is used. If silica excess in the geopolymer system, an adequate source of alumina is needed to form zeolitic products. Following geopolymerization, strong Si-O-Si, Si-O-Al and Al-O-Al bonds result [4]. A study investigated geopolymers based on glass waste obtained by crushing soda-lime glass bottles and class C fly ash mixed in different proportions. Sodium hydroxide solutions with different molarities (0–10 M) were used for activation. The pastes were cured at room temperature. It was found that the glass waste acted as an inert material at the beginning due to the slow reaction speed. After 14 days, as the glass particles reacted with the fly ash, the mechanical strength of the concrete increased. The compressive strength of concrete with 1:3 glass/fly ash precursors, activated with a 5M NaOH solution, was 34.5 MPa [38].

### 2.5. Silica Fume

Silica fume cannot be activated when used alone as a binder because it needs a source of Ca. For example, ordinary Portland cement (OPC) can be used as a source of calcium in the alkaline activation of silica fume. In order to improve the physical and mechanical properties of mortar cement, Caldas and collaborators [39] carried out a study in which they added silica fume to OPC as a pozzolanic additive. The addition of NaOH and KOH had the role of ensuring a combined mechanism of alkaline activation in the silica fume. The rheology of alkali-activated cements differs from OPC rheology due to the loss of workability in alkali-activated mortars and concretes. The partial replacement of cement with silica fume tried to verify its activation potential. The authors replaced OPC with silica fume (10 and 20%) in several ways: without activation, activated by NaOH and activated by KOH. The results showed that NaOH accelerates the setting process of the paste without affecting its workability. It was found that due to the alkaline environment of the cement, the dissolution of KOH, which is also strongly alkaline, is delayed, affecting the workability of the material. The compressive and diametral compressive strengths are higher in samples with silica fume activated alkaline with NaOH and KOH compared to the non-activated material due to the fact that tobermorites and zeolites form by alkaline activation. The addition of 20% NaOH to the silica fume increased the kinetics of reactions. The mechanical strengths of the materials activated with KOH were higher than those activated with NaOH due to the stronger chemical bonds formed [39].

### 2.6. Ceramic Waste Powders

Due to the silico-aluminous crystalline content, ceramic waste can improve the mechanical properties and lastingness of concrete. Many reports in the literature refer to the use of ceramic waste for the partial replacement of OPC, analyzing the mechanical properties and durability of cements in which ceramics are introduced as a binder or fine or coarse aggregate or both. Ceramic waste is known to have pozzolanic properties. Substitution of fine natural aggregates in geopolymers with ceramic waste powders results in lower water absorption. For these wastes, the activation depends on the percentage of Na_2_O, on the SiO_2_/Na_2_O ratio, but also in the hardening techniques. It has been shown that the use of optimal proportions of sodium hydroxide/sodium silicate mixture and the addition of Ca(OH)_2_ is very important for the kinetics of geopolymerization, impacting the mechanical properties and microstructure of geopolymeric mortars. An addition of 4.5% wt Ca(OH)_2_ can lead to good workability and compressive strengths of 21 and 27.5 MPa after 3 and 7 days at 65 °C. Also, with increasing waste content of ceramic powder also increased the temperature resistance of mortars activated with alkalis [40].

## 3. Alkaline Activators

The most common alkaline activators used to obtain geopolymers are sodium or potassium hydroxides (NaOH and KOH) [41] or a mixture of them, sodium or potassium silicates [42,43], carbonates [44], etc. The solubility of the alumina or silica precursors, as well as the structure and properties of the resulting polymers, is influenced by the type of the alkaline activators and their concentration [45,46]. Kale and Chaudhary [47] believed that the size of the cation in the activation solution leads to the formation of a stronger network after polycondensation, being more favored by potassium than by sodium. There are studies that report the influence of the molarity of the alkaline solution on the compressive strength and the setting time of geopolymers. A higher pH value of the alkaline activation solution favors the solubility of the precursors. Geopolymerization is favored by the reactivity of the alkaline solution [48,49,50]. The low-heat Portland cement has a lower shrinkage than medium-heat Portland cement and ordinary Portland cement, due to the degree of hydration owned to alkali sulfates. Cracking of cement-based materials increases as the alkaline sulfate content increases. The contraction of cement-based materials is greater in the presence of K_2_SO_4_ compared to the presence of Na_2_SO_4_, being influenced by the hydration process. The structure and size of the pores, but their distribution, influence the loss of water inside the pores, creating a negative pressure that leads to cracking of the material [51]. Another study [52], regarding fly ash, noted that the mixture of NaOH and Na silicate solution as an alkaline activator led to a higher compressive strength than the use of NaOH alone. To improve resistance, a molar ratio (SiO_2_:Na_2_O) between 1.5 and 2.0 is recommended. An increase in the ratio leads to a decrease in resistance. Purbasari et al. [53] reported that the use of a solution of KOH and water glass as an activator, with additional temperature (60 °C), leads to obtaining a geopolymer with a higher compressive strength. These researchers also observed a difference between the structures of the geopolymers obtained by alkaline activation with a NaOH or KOH solution compared to the geopolymers obtained after activation with a water-glass mixture with either of the two. If in the first case, the geopolymers are more porous, the adding of water glass led to a more dense and homogeneous structure. Giacobello et al. [54] state, that to obtain a geopolymer with certain characteristics, the alkaline activator must be selected according to the nature of the precursors. The silicates used as activators will increase the Si/Al ratio in the precursors, while using hydroxides, as activators, a change to the M_2_O/Al_2_O_3_ and M_2_O/H_2_O ratios, balancing the negativity of the Al tetrahedra and ensuring a pH that favors the dissolution of the precursors before synthesis will occur. The combination of the two types of alkaline activators is considered a good choice because an excess of silicate can inhibit water evaporation, leading to a decrease in compressive strength, and redundancy of hydroxide also leads to a decrease in the geopolymer’s resistance. The structure of the geopolymer can also vary depending on the nature of the used activators. The use of sodium hydroxide, for example, leads to a porous network in the fly ash precursors with Si/Al and Na/Al ratios of 1.5 and 0.48, respectively, in the hydration products. The use of the mixture of NaOH and Na_2_SiO_3_ as an alkaline activator leads to a microporous network between the fly ash particles with Si/Al molar ratios of 2.8 and Na/Al of 0.46. Depending on the nature and ratio of the alkaline activators, they can influence the hydration and microstructure of the geopolymeric material, leading to better or worse performances.

## 4. Fibers Used as Reinforcement of Geopolymers

Materials obtained from natural resources have been used in buildings since the ancient Egyptians who used straw and horsehair for mud bricks. With the focus on reducing environmental pollution to ensure a healthier living climate, new materials reinforced with natural fibers or polymers arose. In the beginning, synthetic fibers were used to reinforce geopolymer composites, such as carbon, steel, glass, basalt [55], organic fibers (polyvinyl alcohol fibers (PVA), polyethylene fibers (PE), polypropylene fibers (PP)), and various carbon or glass fibers [56]. 

Natural fibers can be divided into three categories: vegetable fibers, animal fibers and mineral fibers. Animal fibers are used less due to the collection method, which is difficult. Asbestos, as a mineral fiber, being considered carcinogenic, is not indicated for obtaining ecological materials. Therefore, the most used natural fibers are vegetable ones. In turn, plant fibers can be woody or non-woody. Wood fibers can come from softwood or hardwood. Non-wood fibers are classified according to the part of the plant they belong to: the stem (jute and hemp), fruits (coconut, cotton), leaves (sisal, banana and pineapple), straw (corn, wheat and rice), and grass (bamboo and elephant grass) [57]. Vegetable fibers have two components: cellulose, a linear polymer, made of glucose units, whose concentration depends on the age of the plants, and a structure from a biochemical material called lignin. Cellulose is the main component that influences the mechanical properties of fibers. The degree of cellulose polymerization is different from one plant species to another, and the mechanical properties can also be influenced by the type of fiber, its diameter or length, the extraction method, the harvesting time, the orientation of the fiber, the method used to obtain the geopolymer composite, the porosity, etc. 

The main advantages of vegetable fibers compared to synthetic fibers, in addition to the fact that they contribute significantly to the improvement of tensile and bending resistance, are that the former are found in abundance in nature, are not dangerous, and are biodegradable, light, and cheap. The tensile strength is also influenced by the maturity of the plants, being lower for long fibers than short fibers, most likely due to defects that can appear along the fibers [58]. However, the durability and reinforcement of geopolymers with natural fibers can be damaged due to the degradation of the fibers in the geopolymer matrix. This deterioration can occur both due to external factors and due to internal factors. The alkaline environment of the geopolymers, the fiber–matrix interface, but also the increase in volume of the fibers can influence their deterioration over time. Attacks on natural reinforcing fibers can be physical, biological, mechanical, chemical, or combinations thereof. The degradation of plant fibers is greatly influenced by their composition. The main components being cellulose, hemicellulose and lignin. For example, the arrangement of cellulose microfibrils gives cellulose fibers a good resistance to chemical and biological attacks. On the other hand, the hydrophilic nature of hemicellulose under the action of acids and weak bases can be hydrolyzed and easily degraded at temperatures or following biological attacks [59]. Lignin being hydrophobic is very resistant to most attacks. Practically, the strongly polarized hydroxyl (OH) groups are responsible for the hygroscopic nature of the fibers which leads to their separation in the interface area leading to poor adhesion to the geopolymer matrix. In this sense, solutions are being searched to avoid the degradation of the fibers by carrying out specific treatments, both in terms of geopolymer matrix and fibers [58,59]. One of the tartaring methods, to reduce the water absorption of cellulose fibers, is hornification. This is a technique for modifying the polymer structure of the fiber, through repeated drying and rewetting. The chemical treatment would be another solution. The silanization of the fibers could create much stronger bonds between the fibers and the geopolymer matrix in the interface area, significantly increasing their adhesion. Another treatment could be the heat treatment of the fibers to reduce the OH groups and decrease their hydrophilicity. The carbonation of the cement matrix can also avoid fiber degradation, being a technique capable of reducing the alkalinity of the capillary water, allowing the reaction between CO_2_ and alkaline hydroxides, with the formation of carbonates (calcite), reducing the OH ions in the composite. Adding furnace slag, fly ash, silica fume or metakaolin as pozzolanic agents can be another technique to increase the durability of natural fiber reinforcement. Upon hydration, amorphous Si reacts with Ca(OH)_2_, resulting in a stable hydrated salt of calcium silicate. Matrix pore sealants and fiber impregnation with water-repellent agents is also another technique. All these additional techniques can lead to obtaining the anticipated results by introducing natural fibers for reinforcement in geopolymers [58,59].

## 5. The Mechanism Used to Obtain Geopolymer Cement 

This section may be divided in subheadings. It should provide a concise and precise description of the experimental results, their interpretation, as well as the experimental conclusions that can be drawn.

Pacheco-Torgal et al. [60] proposed a mechanism for obtaining geopolymeric cement based on fly ash. They mentioned that the dissolution of Si and Al takes place through the action of the OH group when the alkaline solution is added to the fly ash, leading to the appearance of the gel following condensation of the higher molecules. The ash particles are attacked first at the surface, penetrating through the defects created on the surface and then from the inside to the surface, until they are almost completely consumed [16]. Following the alkaline activation, the Si-O-Si bonds in the aluminosilicate materials are broken, and new phases are obtained in the solution. Water played the role of a carrier for the activation agent. The penetration of aluminum atoms in the Si-O-Si structure leads to the formation of an aluminosilicate gel in the form of Mn(-(Si-O)z–Al–O)n. wH_2_O, where M = atoms of K, Na or Ca, n = degree of polycondensation, z = 1, 2, 3 or more [18]. The second phases of calcium silicate hydrate and calcium aluminate hydrate can appear depending on the reaction conditions and the composition of the raw materials (if calcium is present in the composition) and water can result from polycondensation. The alkaline activation process is influenced by the concentration of solid matter [18]. Therefore, the strengthening stages of the geopolymeric material can be summarized as: the dissolution of Si and Al atoms, the condensation of the precursor ions into monomers and finally the formation of the polymer structure following polycondensation [16]. Silicon-oxoaluminate polymers are called polysylates. In the polymeric network formed by sialates, positive ions (Na^+^, K^+^, Ca^2+^, etc.) play the role of balancing the negative charge of Al. Si^4+^ and Al^3+^ chains and rings can be formed and cross-linked together by sialated Si-O-Al. Polysialates can range from amorphous to semicrystalline silico-aluminate structures [61]. Davidovits et al. [62] proposed a mechanism starting from class F fly ash (CaO less than 8 wt.%) and using, instead of silicate (K silicate) and hydroxide, an alkali metal silicate together with a slag furnace (Ca/Si ≥ 1). The slag participates as basic Ca silicate in the geopolymeric reaction, and the fly ash particles react only on the surface. Active silica and alumina, on the surfaces of the particles, are obtained after dissolving the ash with an alkali metal hydroxide. Then, there is a polymerization of the active surface groups and the soluble elements, resulting in a gel that will harden, forming the geopolymer [63].

**Figure 4:**
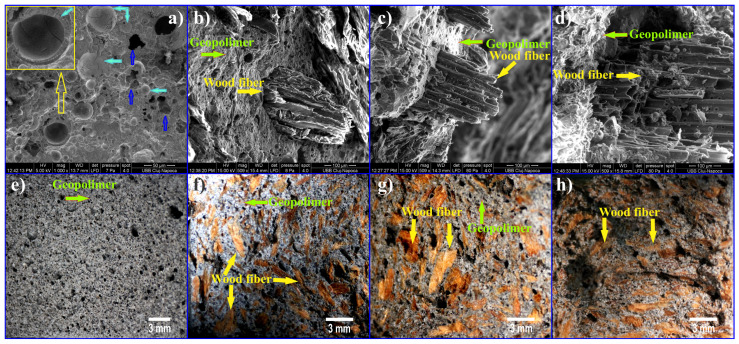
SEM micrographs of fly ash geopolymers: (**a**) 100 wt.% fly ash; (**b**) 90 wt.% fly ash/10 wt.% wood fiber; (**c**) 75 wt.% fly ash/25 wt.% wood fiber; (**d**) 65 wt.% fly ash/35 wt.% wood fiber. Optical image of fracture surface after flexural strength test: (**e**) 100 wt.% fly ash; (**f**) 90 wt.% fly ash/10 wt.% wood fiber; (**g**) 75 wt.% fly ash/25 wt.% wood fiber; (**h**) 65 wt.% fly ash/35 wt.% wood fiber. Composition prepared according to [13].

**Figure 5:**
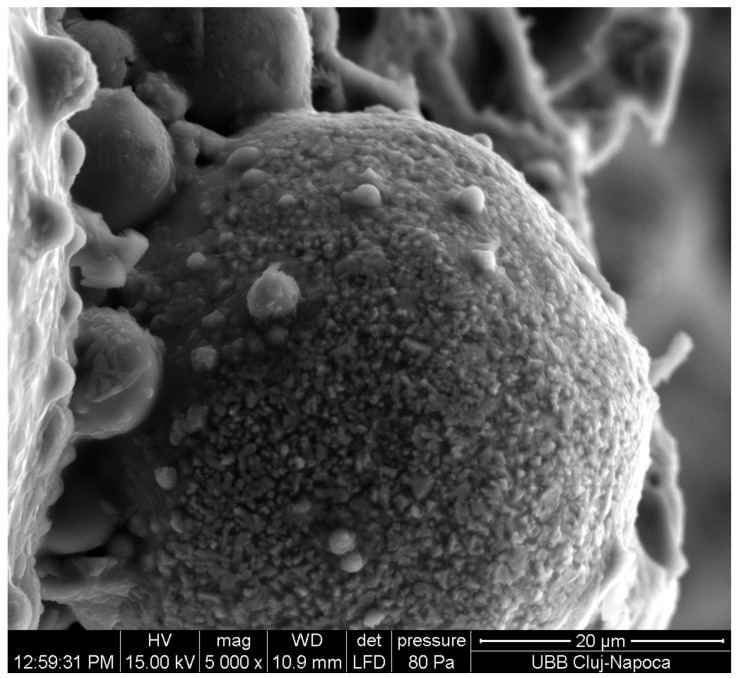
SEM images of the surface of the bonding fly ash in geopolymers. Composition prepared according to [12,13].

When mixing fly ash with the solution of NaOH and Na_3_SiO_4_, spherical particles form that cannot be dissolved, and a layer of gel rich in silica matrix will be formed and cured in time (Figure 4a yellow square, turquoise arrow and Figure 5). These spherical particles from fly ash will contribute to the reinforcement of the geopolymer matrix and will increase the mechanical properties. In Figure 4a, the turquoise arrow shows that some spherical particles of glass are deboned at the fractured surfaces. During mixing the geopolymer paste, air was included, which will remain inside the cured geopolymer (Figure 4a blue arrow). In Figure 4b–d the cured geopolymer matrix around the wood fibers and on the surface of the microfibrils of the wood can be seen. This adhesion between geopolymer-wood fibers is important for mechanical properties, which decrease with increasing quantity of wood fibers [13]. Figure 4f–h shows the distribution of wood fibers in the geopolymer matrix, for the wood fiber reinforced geopolymer composites. The adhesion between the spherical particles and the fly ash silica gel through a network can be seen in Figure 5.

## 6. The Addition of Fibers, Expansion Agent or Polymers in the Building Materials 

Geopolymers with various fibers or polymers could be alternative binders in building materials for improving the properties of masonry. The ratio for precursors and concentration of alkaline activators has an influence on the mechanical resistances, the dry unit weight, and thermal conductivity. Also, the ratios between the added precursors has an influence on the surface structure of the samples. To improve thermal and mechanical properties of light mortars, Tiyasangthong et al. [64] have carried out some tests on cellular mortars with geopolymers based on fly ash reinforced with polyvinyl alcohol (PVA) specimens. The mortars also contained an air foaming agent and an alkaline activator based on silicate and sodium hydroxide (NaOH). The influence of PVA and foam from the composition of the materials as well as the hardening time on the properties of the mortars was investigated. The smallest unit weight (10.10 kN/m^3^) was obtained for mortars with 20 wt.% PVA and 2 wt.% foam. As a result of its high viscosity, a too high concentration of PVA can slow down the geopolymerization reaction, resulting in voids in the material. Therefore, the resistances to compression and flexural strength of the mortars investigated in this study, at all curing times, increased directly proportional to PVA concentrations of up to 5%, after which they decreased. The thermal conductivity of the mortars investigated in the same study, at 7 and 28 days, decreased with increasing concentrations of PVA and foam. The authors believe that due to the larger pores that occur at a higher content in foam, thermal transport is hindered by the formation of larger PVA films [64]. The addition of MgO in the concrete composition can act as an expansion agent, because it forms Mg(OH)_2_ crystals in the presence of water whose growth can reduce the risk of cracking. Wang et al. investigated the influence of MgO percent and its reactivity on air voids, pore structure and concrete permeability. They found that an increase in MgO content and its reactivity reduces the strength of concrete at all hydration ages. Also, the addition of MgO did not have a significant influence on the air voids. With the addition of reactive MgO, the pore structure is compacter at an early age, decreasing the fraction of large capillary pores. With the addition of weakly reactive MgO, the pore structure is compacter at a later age. Therefore, the addition of reactive MgO is beneficial for the impermeability of concrete, because it reacts quickly with water, causing a large expansion. The fractal dimension of the surface or pore volume is related to the permeability of the concrete. The addition of weakly reactive MgO in concrete can lead to an increased strength over time, while reactive MgO cannot [65]. According to another study, the addition of fly ash and PVA fibers increases the compressive strength and tensile capacity of late-age concrete because it refines and optimizes the pore structure. The addition of MgO and shrinkage-reducing additives, on the other hand, reduce compressive and tensile strength at various ages, being less effective in pore refinement [66].

Investigations on other cellular mortars were also carried out by Yoosuk et al. [67], the mortars were composed of geopolymers based on fly ash with a high calcium content, polypropylene fibers (0–3 wt.%), NaOH (2–8 M) and foam (0–2 wt.%). The increase in fiber and foam load and the decrease in the concentration of NaOH led to a decrease in the weight of materials. The lowest value (541 kg/m^3^) was recorded for the sample with the highest fiber and foam load but with 2% NaOH. The highest value of the compressive strength (20.94 MPa) was recorded for the mortar with 0.5 wt.% fibers and 8M NaOH without foam. The maximum flexural strength (3.85 MPa; density of 1279 kg/m^3^) was recorded for the sample with 2.5 wt.% fibers, 8M NaOH, without foam. For the samples with 2M NaOH (a more desirable concentration from an environmental but also an economic point of view), the maximum values of the compressive and bending strengths were 12 MPa and 2.07 MPa, respectively, corresponding to the requirements of the standard [67]. Tabyang et al. [68] investigated the maximum dry unit weight, and mechanical properties, and performed a microstructural analysis for lateryl soil samples reinforced with coconut fiber and mixed with a fly ash-based geopolymer. As an activation solution, they used a mixture of sodium silicate (Na_2_SiO_3_) and NaOH. The authors believe that the optimal precursor/sample mixture is given by the following ratios: 1/1 lateryl soil/geopolymer with fly ash, 80/20 Na_2_SiO_3_/NaOH (using 5 M NaOH) and a content of 0.5 wt.% coconut fiber. It was shown that coconut fiber promoted the geopolymerization reaction by the fact that the matrix of the sample with coconut fiber was compacter than that of the sample without coconut fiber. However, a larger addition of coconut fiber led to an increase in the porosity of the material. Along with the increase in the ratio lateryl soil/geopolymer with fly ash and the concentration of NaOH, the maximum dry unit weight of the samples also increased. It was also found that the alkaline activator content of the geopolymer with ash is constant when the NaOH concentration is higher. After 7 days, the unconfined compressive and flexural strengths increased with the addition of 5 wt.% coconut fiber [68].

## 7. The Influence of Different Precursors on Geopolymer Properties

The reintroduction of geopolymers in the manufacturing process of new building materials represents a real challenge for the future, both for environmental protection and for the huge stocks of waste from demolitions or the remains left after their use. Saving the energy consumed to obtain geopolymers, but also the reduction in gas emissions, represents their notable advantages that could lead to the replacement of some common binders such as cement or lime [69]. Wattimena and collaborators [70] studied the replacement of cement as a binder in conventional concrete, with fly ash as a source of aluminosilicates. They investigated several types of fly ash, obtained in different ways, to observe their influence on the properties of the obtained geopolymers. It was concluded that different sources of ash can influence the synthesis of geopolymeric concretes. The authors conclude that the chemical composition and the physical properties of the fly ash influence the behavior of the paste and the reinforced geopolymer. Also, for a low Ca content (class F ash), fewer problems appear compared to those for ash with a higher Ca content, where the reaction time is faster and intermittent. However, geopolymers with higher Ca content can provide compressive strengths over time, due to the hydration reaction in addition to the polymerization reaction. Because the properties of fly ash cannot be controlled, the authors say that the fly ash must be further tested before being used as a source of aluminosilicates in geopolymer. Jiang et al. [71] followed the effect of temperature (up to 1200 °C) on the thermo-mechanical properties of geopolymers obtained from class C and class F fly ash. They noted that geopolymer pastes with class F fly ash presented higher mechanical properties and thermal performance at temperatures below 500 °C, while others, from class C fly ash, recorded higher mechanical properties and superior thermal performance at higher temperatures (>800 °C).

Kirgiz [72] carried out a study in which he proposed to overcome some effects such as rapid gelation and reduced initial strength of fly ash substituted cement of class F by the addition of nanographite particles and of superplasticizer. The pastes were composed of lime with nanographite and water and the variation in the Ca(OH)_2_ content was monitored. In addition to sand, the investigated mortars had these cements in their composition. In some compositions, only water was added; in others, a superplasticizer was added. The author emphasized the role of nanographite as a consumer of the Ca(OH)_2_ content. Improvement in the mechanical resistances was achieved with the help of nanographite, reducing the amount of pure Portland cement. The superplasticizer reduced the rapid gelation process.

In their study, Wang et al. [73] obtained geopolymers from alkaline-activated metakaolin with a mixture of hydroxide and Na silicate. The Na/Al ratio gradually increased in the samples from 0.43 to 0.93 and the mechanical properties increased until Na/Al = 0.73 ratio. Once the Na/Al molar ratio was fixed, the Si/Al ratio also changed, from 1.7 to 1.95. They found that the highest mechanical strength value (21.3 MPa) was recorded for a Si/Al ratio of 1.90 and the strength of the geopolymer is mainly based on the formation of the NASH gel. The moderate increase in alkalinity led to a faster dissolution of metakaolin, positively influencing geopolymerization. However, a higher increase can inhibit the polyreaction through high crystallization. Another finding was related to the water content, which was similar for all samples up to 20 days after hardening and finally reached 15%. The optimal mixing ratio differs from material to material, depending on the source of aluminosilicate used and other external factors [73].

Fly ash geopolymers can improve soil resistance; due to their high dry weight and low plasticity index, the lateritic soil can be used in paving materials [1]. Tesanasin et al. [1] carried out a study with the aim of improving the properties of the marginal lateritic soil, with the help of a one-part fly ash geopolymer with high calcium content. This method uses the alkaline activator in solid form to ease the production process and avoid some shortcomings, such as low workability or corrosion. The precursors are mixed, and water is added only at the end. As an alkaline activator, they used NaOH flake form, adding different concentrations of solid activator to the composition of the samples. They found that to improve unconfined compressive strength, indirect tensile strength and microstructure, the optimal activator content is 20 wt.% or less. After 7 days, the unconfined compressive strength was 1930 kPa under soaked conditions and 2800 kPa under unsoaked conditions, while the indirect tensile strength was 270 kPa and 400 kPa. The values of both resistances were lower in the case of soaked samples because of the repulsive forces between the clay particles. After 28 days, the values of both mechanical resistances increased, which means that the reaction between the precursors continued during this time interval. Furthermore, the Ca content influences both the setting time and the mechanical properties. From the microstructural analysis of the sample surfaces, was observed that when a higher content of alkaline activator is added, beyond the optimum, the appearance of microcracks can lead to a weakening of the microstructure [1].

Suksiripattanapong et al. [74] carried out a study on the possibility of improving soft Bangkok clay (watery soil with low shear resistance and compressibility), through prefabricated vertical drainage, grouting or deep mixing of the soil. They investigated the use of geopolymers with a PVA and fly ash with high Ca content to improve the resistance of this type of soil. As an alkaline activator, they used a liquid mixture of sodium silicate Na_2_SiO_3_ and NaOH. The authors [74] investigated the influence of the content of PVA, fly ash, water, and alkaline activator, but also of the ratio between the two components of the activator, the added concentration of PVA and the curing time on the microstructure of the sample surfaces and on the unconfined compressive strength. It was found that after 28 days, the unconfined compressive strength of 1026 kPa was obtained for a ratio between the two components of the alkaline activator equal to 1, an ash content of 40 wt.% and an activator/fly ash ratio equal to 0.6. An improvement in resistance was observed by 40–42% (both after 7 and after 28 days), due to the formation of strong bonds between the soil particles, by adding a content of 15 wt.% PVA, with a concentration of 4 wt.%, thus meeting the requirements of resistance. From the microstructural analysis, was observed that a lower content of PVA (5 wt.%) seeps into the pores of the geopolymer with fly ash, while a higher content (25 wt.%) surrounds the ash particles, delaying the geopolymerization. Therefore, the PVA films found in geopolymerization products were observed with an optimal content of 15 wt.%. Another finding of the study was that the unconfined compressive strength of the fly ash geopolymer increases over the 28 days for a Na_2_SiO_3_/NaOH activation mixture ratio < 2, by consuming the silica and alumina content of the fly ash leading to the formation of geopolymerization products over time. In the case of a Na_2_SiO_3_/NaOH ratio > 2, the increase in resistance over time is slower, due to the slower geopolymerization process, due to the lower NaOH content. After 7 and 28 days, respectively, the unconfined compressive strength of the samples increased to an activator/ash ratio of 0.6 as the ratio of the activator components Na_2_SiO_3_/NaOH decreased and the water content decreased. Above this value, the resistance increases as the ratio of the activation components increases. The NaOH content in the activator is important; if it is high, even for a lower Na_2_SiO_3_/NaOH ratio, it leads to the appearance of large pores in the samples where the fluid can drain, slowing down the geopolymerization process [74].

Slag-based geopolymers have been included in building materials since 1957 [75]. Ground granulated blast furnace slag contains oxides of calcium, silicon, aluminum, and magnesium [76]. Zhuguo and Sha [77] analyzed the carbonation depths of some geopolymeric concretes, using fly ash and ground furnace slag as an aluminosilicate substrate, conducting the carbonation test after different periods of time. The study concludes that at room temperature, the resistance to carbonation of geopolymeric concrete based on a mixture of ash and slag is lower than that of ordinary concrete with Portland cement. The increase in resistance to carbonation is very much on the blast furnace slag. The resistance in carbonation results in the increase in the slag content from the active filler mixture and the NaOH concentration of an active activator, with a decrease in the ratio between the activator and the active filler but also from the ratio between the water content and the active filler. Furthermore, an increase in hardening temperature and fineness of the slag contributes to ensuring greater resistance to the carbonation of geopolymeric concrete. Aziz et al. 2020 [76] performed a series of compressive strength tests on some geopolymers based on granulated and ground blast furnace slag using different ratios of solid/liquid and alkaline activators and came to the conclusion that at a solid/liquid ratio equal to 3 and an alkaline activator ratio of 2.5, after 28 days of curing, a compressive strength of 168.7 MPa was obtained. Furthermore, the formation of tobermorite and CaCO_3_ (confirmed by XRD), due to higher concentrations of Ca in areas with silica and alumina, contributed to the increase in compression resistance.

## 8. Conclusions

Based on the literature, the most used precursors for geopolymer are fly ash, metakaolin, glass waste, and blast furnace slag. The compressive strength of geopolymer increased when the particle size of class F fly was the smallest and the activator/fly ash ratio was the highest. F-type ash is recommended in the synthesis of geopolymers. The more aluminum in the aluminosilicate source (at least 20 wt.%), the higher the reactivity. The mixing method of geopolymers also has an important impact on the setting time. The most used activators are NaOH, KOH and Na_2_SiO_3_. The mechanical properties can also be influenced by the type of fiber, diameter, length, extraction method, harvesting time, orientation of the fiber, the method used to obtain the geopolymer composite, and porosity. Geopolymers with various fibers or polymers could be alternative binders in building materials for improving the properties of masonry. The addition of MgO to the concrete composition can act as an expansion agent and shrinkage-reducing additive; on the other hand, it reduces compressive and tensile strength at various ages, and is less effective in pore refinement. There are multiple possibilities of using geopolymers to support a circular economy, by transforming some waste into new materials with the potential of reusing them in various industries. Most of the limitations of the literature studies are related to the durability, short-term and long-term mechanical properties of the geopolymers, as there is still no well-established code of practice regarding the composition and optimal ratio of components in geopolymers or of the use of geopolymers, taking into account the setting reactions involved.

## Data Availability

The data that support the findings of this study are contained within this review.
